# Configuring the PrEP user: framing pre-exposure prophylaxis in UK newsprint 2012–2016

**DOI:** 10.1080/13691058.2020.1729420

**Published:** 2020-04-07

**Authors:** Ingrid Young, Nicola Boydell, Chris Patterson, Shona Hilton, Lisa McDaid

**Affiliations:** a Centre for Biomedicine, Self and Society, Usher Institute, University of Edinburgh, Edinburgh, UK; b Centre for Research on Families and Relationships, Usher Institute, University of Edinburgh, Edinburgh, UK; c MRC/CSO Social and Public Health Sciences Unit, University of Glasgow, Glasgow, UK

**Keywords:** Pre-exposure prophylaxis, HIV, media, gender, health inequalities

## Abstract

Pre-exposure prophylaxis (PrEP) has been hailed as a revolutionary intervention for HIV prevention. PrEP’s controversial status in the UK has generated significant media coverage. It is important to understand what role the media plays in framing PrEP policy issues. We undertook a qualitative analysis of UK newsprint articles between 2012 and 2016 to examine how PrEP was framed as a public health intervention up until a controversial policy decision not to provide PrEP in England. We identified how scientific evidence was deployed to shape two narratives: *ir/responsible citizens* focused on imagined PrEP users and their capacity to use PrEP effectively; and *the public health imperative*, which described the need for PrEP. Our analysis demonstrates the particular ways in which scientific evidence contributed to the certainty of PrEP as an effective intervention within UK newsprint. Scientific evidence also played a key role in framing PrEP as an intervention specifically for cis-gendered gay and bisexual men, playing into wider debates about who is a deserving patient and the appropriate use of public resources. Practitioners in the UK and elsewhere should be aware of these constructions of the PrEP user to ensure equitable access to PrEP beyond gay and bisexual men.

## Introduction

In November 2016, the Court of Appeal of England and Wales ruled in favour of a judicial review brought forward by the National AIDS Trust (NAT), a UK HIV policy organisation (Court and Tribunals Judiciary 2016). NAT argued that the provision of pre-exposure prophylaxis (PrEP) – the use of existing HIV treatment in the form of a (usually) daily pill by HIV-negative individuals to prevent the acquisition of HIV – *was* within National Health Service (NHS) England’s remit of provision (NAT 2016). The ruling ended a very public court battle challenging NHS England’s controversial March 2016 policy decision that funding PrEP did not fall within its remit. This public battle garnered significant UK media attention and public debate ensued concerning the NHS’s role in funding a drug to prevent HIV amongst gay and bisexual men. Grounded in claims that funding this expensive drug would take away from essential medicines for more worthy patients (e.g. children, cancer patients), the issue focused squarely on the high cost of a lifestyle – and not life-saving – drug that was seen to benefit a minority population (gay and bisexual men). The implicit messages, reinforced by homophobic undertones reminiscent of early AIDS reporting (Watney 1987), were that gay and bisexual men should take responsibility for their own health through condom use and behaviour modification rather than relying on expensive, publicly-funded pharmaceuticals (Twocock 2016; Duffy 2016; Mowlabocus 2019). Although the ultimate legal resolution determined that responsibility for commissioning PrEP lay with NHS England – which would come with a new set of controversies – the debate centred around both who PrEP was for and the need for PrEP itself.

Media reporting on biomedical developments allows us to examine how scientific evidence itself is deployed, translated and appraised in public spaces. As we have seen throughout the history of HIV, mass media institutions have played ‘a critical role in shaping how scientific controversies are interpreted, and adjudicated’ (Epstein 1996, 22). In her work on AIDS as a biomedical and a cultural phenomenon, Paula Treichler describes how multiple meanings of what we would now term HIV originate, proliferate and take hold, arguing

we need an epidemiology of signification – a comprehensive mapping and analysis of these multiple meanings – to form the basis for official definition that will in turn constitute the policies, regulations, rules and practices that will govern our behaviour for some time to come … as we have seen, these may rest on ‘facts’, which in turn may rest on the deeply entrenched cultural narratives (Treichler 1999, 39).

While the scientific evidence for PrEP, including clinical and cost-effectiveness, may have been clear to experts prior to the NHS England policy decision being taken (Lancet 2016), it is important to understand how evidence was deployed and shaped by wider cultural narratives, and to consider the specific role of media in framing these UK-wide health policy issues. In this paper, we examine how UK newsprint media reported PrEP as a public health intervention prior to the NHS England decision. While the media coverage of PrEP after March 2016 was steeped in questions around sexuality, responsibility and entitlement, we ask how PrEP was configured in newsprint media as a public health intervention up until this point and consider how this may have shaped subsequent public PrEP debates.

## Framing public health narratives in newsprint

The media and its engagement with science and related health research play a key role in shaping public understandings of health (Seale 2002). Indeed, there is long history of critical analysis focusing on the cultural politics of HIV and AIDS, and the role of the media in representing and constructing understandings of the HIV epidemic, as well as shaping policy and public health endeavours (Epstein 1996; Treichler 1999; Watney 1987). The role of newsprint in particular as emblematic of mass media, and how it frames health narratives, has been well established in the social science literature (McCallum and Holland 2017; Pickersgill et al. 2017; Williams et al. 2008; Hilton et al. 2010).

In this paper, we examine newsprint media as a discursive site, recognising the role it plays in shaping understandings of public health and related technologies, but also in how it engages more directly with wider public health and/or scientific imaginaries (Davis 2014). Like Pickersgill et al. (2017), we explore how newsprint articles ‘imagine and articulate’ understandings of science within a UK context. It is important to note that this relationship – between media and publics – is mediated by the ways in which media, scientific, health and community stakeholders interact (Briggs and Hallin 2016; McCallum and Holland 2017). In their book *Making Public Health*, Briggs and Hallin (2016) draw on the concept of biomedicalisation (Clarke et al. 2010) to describe how media and public health actors interact with each other in an increasingly technoscientific era. At a time when media outlets commit fewer resources to producing science- or health-based stories (Stansistreet 2015), public health and research institutions are increasingly investing in media specialists to communicate and tailor research findings for wider publics, including media outlets. Biomediatisation, Briggs and Hallin argue, results in a shift from reporting to forms of co-producing news stories. Moreover, as these new mediated communications relationships adapt to ‘the logics and routines of news media to get their message across, they may gloss over the complexities and contingencies of scientific knowledge’ (McCallum and Holland 2017). For the purposes of this paper, it is imperative therefore to consider not only who is a part of creating these public health narratives, but on what evidence they are based and how this evidence is deployed to *produce* PrEP itself. In other words, the very nature of what PrEP is as an intervention is constituted through these configurations of evidence (Rosengarten 2010; Montgomery 2012).

This paper also considers how the technology *user* is shaped in or through how the technology itself is imagined or conveyed (Montgomery 2012; Johnson 2017). Oudshoorn et al. argue that

‘a semiotic approach to user-technology relations enables us to analyse how, even in cases where users are not formally involved in the design, technologies may become adjusted to certain groups of users because of the incorporation of specific images of the future users’ (Oudshorn, Rommes, and Sinestra 2004).

That is, the end user of a particular technology, including a pharmaceutical intervention, is already imagined and shaped throughout the design and/or implementation of the technology itself. However, the user and technology interact with and reconfigure each other to co-constitute technologies. As a result, we need to consider the ways in which end-users are imagined and re-imagined as technologies are deployed (Montgomery 2012). We draw on and add to the work of Holt, who explored how the PrEP user was configured in an earlier PrEP era, through scientific writing (Holt 2014). Holt focused on how PrEP was configured specifically for gay and bisexual men through research trial products (e.g. articles, testimonies). While Holt looked primarily at US research, we consider the imagined use of PrEP as told through UK newsprint, paying particular attention to who the PrEP user might be, and if and how they may be gendered (Johnson 2017; Montgomery 2012).

In exploring PrEP and its imagined users in UK newsprint, we also engage with the wider biomedicalisation of HIV (Young, Flowers, and McDaid 2016). The increasing move to biomedical HIV prevention interventions globally has not been without controversy. Opposition to biomedicalisation of HIV prevention related not only to concerns around costs of pharmaceuticals and fear of technologies, but also the erasure of social practices which have been instrumental in maintaining prevention (and care) practices throughout the HIV epidemic (Kippax and Stephenson 2012; Nguyen et al. 2011). Understanding how scientific evidence of efficacy and effectiveness is deployed within this contested space is critical. Throughout the epidemic, scientific evidence has been used to shape particular ends and has itself been shaped by social norms and values (Epstein 1996; Treichler 1999). Paying attention to how and where scientific narratives of health and prevention emerge in relation to PrEP is also about exploring how PrEP is framed in relation to wider histories of social and health inequalities.

## Methods

We employed a qualitative content analysis approach by undertaking thematic analysis of the latent content of newspaper articles (Altheide 2012). Latent content here refers to the underlying meaning of texts, which demands an interpretative coding approach (Neuendorf and Kumar 2006). We selected 16 mainstream, UK national daily and Sunday newspapers with high circulations (Press Gazette 2016), and four Scottish national newspapers, representing a broad range of newsprint readership profiles: *The Daily Mail* and The *Mail on Sunday*; *The Daily Telegraph* and The *Sunday Telegraph*; *The Express* and *The Sunday Express*; *The Guardian* and *The Observer*; *The Independent* and *The Independent on Sunday*; *The Mirror* and *The Sunday Mirror; The Times* and *The Sunday Times*; *The Sun*; and *Metro*; *The Herald* & *Sunday Herald*; *The Scotsman* & *Scotland on Sunday*; *The Daily Record and Sunday Mail*; *and The National*. The Sunday counterpart of *The Sun* was not archived in the Nexis database used, and *Metro* does not have a Sunday counterpart.

This sampling frame includes publications from different newspaper ‘genres’ (tabloid, middle-market tabloid and serious), employing a typology used previously (e.g. Hilton et al. 2010; Patterson, Hilton, and Weishaar 2016) to ensure a sample of publications representing a demographically diverse range of newsprint reading audiences. Content included material available through online newsprint coverage.

Our search period started on 1 January 2012, the year that PrEP was approved by the US Food and Drug Administration (FDA) and covered a period during which PrEP became recognised globally as a viable HIV prevention tool. We ended our search on 31 March 2016, the point at which NHS England announced their decision not to fund PrEP. This end point allowed us to focus on how PrEP was configured as an intervention through the media before debates moved more explicitly towards issues of entitlement. Using the Nexis database, we searched for articles that either contained ‘HIV’ and/or ‘AIDS’ within five words of the term ‘pill’ or contained one or more of the following terms: ‘Pre-exposure prophylaxis’; ‘Truvada’; ‘anti-aids’; ‘anti-HIV’; ‘HIV drug’ and ‘AIDS drug’. All articles that matched the search string were downloaded and subjected to initial review. This yielded 156 articles. To ensure that only articles predominantly reporting on PrEP were included, any articles of which more than 50% of the text was not relevant to PrEP were excluded. We excluded 109 articles at this stage resulting in a sample of 47 articles for full review.

All articles were initially read by the first and second authors to generate broad thematic categories (Table 1). Articles were systematically read and coded according to these thematic categories, including recording general interpretative notes about each article, by authors one, two and three. At this stage, three more articles were excluded, providing a final sample size of 44 (see Table 2). Only two articles came from Scottish newspapers, with the remaining 42 from mainstream UK national newspapers. No separate analysis was undertaken of the Scottish articles due to small numbers. To promote consistency, one quarter of the articles were double-coded, and coders discussed their interpretations of the thematic categories throughout. Once coding was complete, authors one, two and three employed both a deductive and inductive approach to analysis by reflecting on article coding, but also drawing on a grounded theory approach to consider which stakeholders were included in the articles, what was reported, and how it was presented. Through multiple, iterative discussions, two broad narratives and a timeline of key PrEP narrative events were identified.

## Findings

Although our search period covered more than 3 years (2012–2016), we found relatively few newsprint articles on PrEP. We also found very little difference in tone or coverage between different genres of newspaper. Reporting during this period was largely in response to the publication of UK and international scientific findings, conference presentations and/or policy decisions. In particular, our analysis identified the release of results from *PROUD –* a UK based PrEP Randomised Control Trial (RCT) with gay and other men who have sex with men (men who have sex with men) in England - as a central scientific event in patterns of reporting in UK newsprint. It was possible to identify two distinct time periods in PrEP reporting: *Priming for PROUD*, 15 articles from July 2012 to October 2014 and; the *Post*-*PROUD* period from February 2015 to the end of March 2016, 29 articles, two-thirds of the sample. The beginning of the *Post*-*PROUD* period, which followed three months in which no reporting on PrEP was identified, began with reporting on the presentation of UK PrEP trial *PROUD* findings at the International Conference on Retroviruses and Opportunistic Infections (CROI), a key HIV scientific conference. From February 2015 onwards, reporting substantially changed in focus and tone.

We also identified two broad narratives. The first narrative, *ir/responsible citizens*, focused on PrEP users and their capacity and responsibility to use PrEP correctly, which framed the un/certainty of PrEP as an effective intervention. The second narrative, the *public health imperative*, described if and why PrEP might be needed as a public health intervention. There was a marked difference in both themes in the two time periods (see Table 3).

### Ir/responsible citizens: priming for PROUD

During this period, imagined PrEP users and their sexual practices were presented as a source of unknown or increased risk were PrEP to be made available. Descriptions focusing on potential reduction in condom use and its assumed related increases in harms (e.g. increased risk of HIV and STIs) characterised PrEP users as irresponsible. While not always the dominant message, this narrative was consistently present. Regular, ongoing constructions of irresponsible PrEP users were juxtaposed with, and in some cases part of, expert opinion and reporting on scientific findings. Familiar stereotypical language (Watney 1987) used to paint PrEP users as irresponsible citizens included ‘unsafe sex’ when describing condomless sex, calling PrEP a ‘party drug’, and describing ‘increased risk taking’, ‘promiscuity’ and/or a ‘reduction of condoms’.

Voices from community opposition to PrEP were used to air many of these concerns. Quotes evidencing opposition to PrEP came mainly from the AIDS Healthcare Foundation, a US organisation known for strong opposition to PrEP, with its founder Michael Weinstein by far the most quoted voice. Weinstein and other community voices argued that the provision of PrEP would lead to a ‘false sense of security’ (Daily Mail, 17 July 2012) and threaten condom use, the most reliable prevention measure against HIV. The irresponsible citizen narrative highlighted specific problems which would cause the ‘failure of PrEP’. In many articles, PrEP failure was characterised by poor adherence, in which people were imagined not to take PrEP regularly because of forgetfulness or side effects. However, the dominant failure comprised forms of ‘risk compensation’, whereby PrEP would facilitate an increase in STIs (and possibly HIV) because of reduced condom use, and an assumed change in sexual practice to increased sexual partners and ‘risky behaviours’.

In some articles, key UK scientific or community HIV experts refuted or tempered the ir/responsible characterisation of the imagined PrEP user. While Professor Sheena McCormack, head of the UK *PROUD* trial, expressed concern about encouraging risky sexual behaviour, she was also reported as providing examples or situations in which condom use might not be possible, and explaining how PrEP could be about ‘helping people to be responsible’ in these cases (The Times, 17 October 2014). Similarly, Justin Harbottle from a well-known HIV charity, Terrence Higgins Trust (THT), described fears around PrEP as ‘misplaced’, and compared PrEP to the contraceptive pill, highlighting the need for choices of prevention method (Independent, 17 May 2014). In spite of these tempered comments, reporting in most articles cast doubt on the capacity or willingness of PrEP users to act responsibly and use PrEP correctly, thus potentially undermining PrEP’s effectiveness to prevent HIV.

### Ir/responsible citizens: *post-PROUD*

With the presentation of *PROUD* results in February 2015, there was a noticeable shift in the representation of imagined PrEP users. In contrast to the sustained focus on the potential failure of PrEP users in the earlier period, specific mention of PrEP users and their capacity to use PrEP was largely absent. Instead, comment was offered on the use of PrEP and ongoing use of condoms, suggesting the capacity of PrEP users to act ‘responsibly’.

A study of British men showed that they were willing to take the pill, which did not appear to encourage unsafe sex …. It is allowing individuals to negotiate what sort of sex they want. It provides them with protection. (The Times, 25 February 2015)

Although articles continued to refer to condomless sex as ‘unsafe’, there was a marked absence of reporting on the potential for ‘irresponsible’ sexual practice. One article, reporting the *PROUD* study findings published in The Lancet, addressed these previous concerns directly.

A key concern has been that Truvada would become a ‘party drug’, used instead of condoms to prevent infection, thereby having the unwanted side-effect of increasing the risk of passing on other sexually transmitted infections. But this, it turns out, has not been the case – there was no significant increase in other STDs recorded during the Lancet trial. (Independent, 11 September 2015)

Other articles, while not refuting a previous stance outright, used more neutral language in their reporting.

Since PrEP does not work all the time, nor does it prevent sexually transmitted infections like syphilis and gonorrhoea, health authorities say people should continue to use condoms regularly. (Telegraph, 24 February 2015)

Instead of focusing on specific users, articles reminded readers that PrEP did not prevent STIs, rather than undermine PrEP as an intervention itself.

A small minority of articles raised the issue of the potential of decreased condom use and increased STIs if PrEP were made available, but this was often tempered by, or refuted with, evidence from the *PROUD* study:

Men taking part in the study reported they did not change their condom use because of PrEP – evidence that was borne out by the fact that infection rates of other STIs were similar in both groups. (Independent, 25 February 2015)

Of the 29 articles from this period, there were two notable exceptions to this portrayal of imagined PrEP users. Similar to the irresponsible citizen theme in the earlier period, two articles described concerns around promiscuity or ‘riskier sex without condoms’ (Daily Mail, 26 February 2015). Both reported and asked directly if PrEP would ‘encourage risky sex’ (The Times, 14 January 2016), with one article referencing US criticisms. However, both articles also reported evidence from *PROUD* showing no increase in STIs between the PrEP and deferred arms of the study. The tone of these articles was more negative, as the articles’ structure diminished this evidence, focusing instead on potential risks of PrEP. The tone and emphasis of these articles, however, were not in keeping with most articles from this time period.

Voices from community activists and PrEP users were largely absent during this period. This meant that although Weinstein’s opposition was no longer present, neither were there community voices discussing the potential for PrEP. This scientific discussion contrasted with the narratives around PrEP users present in the earlier period. The absence of comment on, or tempered representation of, potential risks from PrEP – thanks in large part to the *PROUD* findings – resulted in a diminishing of the irresponsible citizens narrative and a shift towards a potentially effective intervention itself.

### Public health imperative: priming for PROUD

Articles in the early period consistently provided general estimates of HIV diagnoses in UK, USA or globally. Descriptions included total numbers of HIV diagnoses and expected yearly diagnoses. Some, especially of the UK epidemic, also referenced the number of people estimated to be undiagnosed.: ’… 96,000 people in the UK are currently living with HIV. Of these people, 22,600 are thought to be unaware of their infection’ (Daily Mail, 13 June 2013). Some articles went on to identify groups most at risk of, or affected by, HIV. This often included a list of groups including gay and bisexual men, people in relationships with someone living with HIV and people who inject drugs.

Of the 6280 new cases of HIV in 2011, around 48% were infected through heterosexual sex, and 48% through sex between men …. The CDC now estimates as many as 275,000 uninfected gay men and 140,000 heterosexual couples, in which one partner is HIV-infected, could benefit from PrEP. (Daily Mail, 17 May 2014)

With the announcement in October 2014 that the *PROUD* trial would be offering PrEP to all participants, including those on the deferred arm, the presentation of epidemiological evidence shifted noticeably. Articles began to include information about HIV rates which more obviously focused on the specific benefit of PrEP for gay and bisexual men.

Nearly 100,000 people are living with HIV in the UK. Sex without a condom is the most common means of infection. Nearly half of all new infections are among men who have sex with men. (Independent, 30 October 2014)

Some articles addressed the cost of PrEP as they explored the need for the intervention. Available at the time only as patented drugs, PrEP was reported as very costly. One article outlined that ‘the drug is likely to raise questions over its high price’ (The Times, 10 February 2014), indicating that it may not be a cost-effective intervention. Other articles towards the end of the period outlined similar concerns but indicated that there may be ways around the normally prohibitive pricing.

If the final results are good, public-health experts hope the drug could be made available on the NHS to those at high risk, although negotiations would have to take place with manufacturer Gilead to find a price the health service can afford. The drug can cost the equivalent of £7500 a year in the US. (The Guardian, 18 October 2014)

High cost combined with a more general framing of HIV as a public health issue, rather than for a specific group, presented uncertainty in PrEP as a viable intervention, ready for implementation.

### Public health imperative: *post-PROUD*

By February 2015, and the announcement of *PROUD* findings at CROI, the presentation of any HIV context in the articles now focused almost entirely on gay and bisexual men in the UK. Articles included specific information on existing HIV rates amongst this group, and the potential numbers of gay men who could benefit from PrEP.

Men who have sex with other men are most at risk of HIV, accounting for nearly half of the 6000 new cases each year. One in 26 gay men in the UK estimated to have the disease, rising sharply to one in eight in London. (Daily Mail, 14 January 2016)

Noticeably absent from most articles were references to other groups for whom PrEP might be helpful. This was in stark contrast to articles from the earlier time period. There was no mention of trans individuals and communities in spite of their being disproportionately affected by HIV (Baral et al. 2013). We note, therefore, that references to gay and bisexual men were largely, if not exclusively, made with cis-gendered gay men in mind.

Exceptionally, a very small number of articles from this time period did not present PrEP as a public health need in the same way. Two articles from the Times continued to provide a broader HIV context. One, which reported the modelled effects of PrEP on HIV infection rates amongst gay men in the UK, raised the need for attention to ‘other at-risk groups’:

[…] if all gay men were offered it, infection rates would fall by 59%, preventing 10,000 cases of HIV by 2020. Even if it were limited to the quarter of gay men who have more than one new sexual partner a year, 7400 cases would be prevented …. What about women and others at risk of HIV? Women with an HIV-positive partner are likely be included. Gay men will be prioritised because they are high-risk, easy to identify and those coming to clinics are keen to protect themselves. Evidence of effectiveness is also stronger. Other at-risk groups, such as some African communities and drug users, will be considered later. (The Times, 14 January 2016)

A third article, which described new modes of PrEP delivery, such as vaginal rings and injectable PrEP, described how ‘vaginal transmission accounts for the majority of new HIV infections across the world’ (Daily Mail, 21 March 2016).

The public health imperative theme during this period also drew on cost to frame PrEP certainty and need. Whereas earlier reporting focused on the expense of PrEP, reporting here drew on the cost-effectiveness of PrEP as an intervention to prevent HIV. Articles acknowledged ongoing high costs of PrEP, but described how such an effective intervention, if used by the most appropriate PrEP users, would result in longer term savings to the NHS.

[…] it will have to consider whether the pills, which will cost £423 per month for each patient, will be cost-effective, and what the criteria should be for accessing them … If we can stop people getting HIV by giving them PrEP, we have an ethical duty to do so. Furthermore, over the course of their lifetime the treatment of those 19 men will cost the NHS nearly £7m. So the financial argument is clear, as is the ethical one. PrEP needs to be available on the NHS as soon as possible for all those who need it. (Independent, 25 February 2015)

The imperative for PrEP was also captured in specific language used by public health experts. Articles frequently and repeatedly drew on THT Medical Director Dr. Michael Brady’s description of PrEP as a ‘game-changer’, encapsulating the need for PrEP as a significant tool with which to combat the current, and urgent, HIV epidemic amongst gay and bisexual men in the UK. With such an effective intervention that would save the NHS money in the longer term, the public health imperative to implement this ‘game-changing’ intervention was clear.

## Discussion

In an era of increasing pharmaceutical prevention-based policies and the need to understand what shapes policy implementation, our analysis illustrates how specific forms of scientific evidence about PrEP were deployed within UK newsprint, playing an important role in the configuration of imagined PrEP users. Coverage during this period mobilised scientific evidence, first to characterise PrEP as an uncertain intervention in the context of an ongoing, global epidemic and, ultimately, to frame PrEP as much needed, ‘game-changing’ public health intervention specifically for gay and bisexual men. Reporting initially queried the capacity of individuals to use PrEP appropriately, drawing on well-rehearsed stereotypes to suggest that irresponsible sexual practice would threaten the effectiveness of the intervention. However, *PROUD* trial results were used to show that PrEP users had the capacity to act as responsible citizens (Young et al. 2019). This shift in reporting and diminishing doubt about PrEP use added weight to the certainty of PrEP as a feasible and effective intervention within these UK media narratives.

The use of community and scientific voices was key in the framing of evidence (Briggs and Hallin 2016). The inclusion of community voices and some opposition in the early period framed PrEP as an uncertain intervention. This opposition – along with most community voices – disappeared from newsprint sources with the success of *PROUD*, as the role of scientists, clinicians and policy makers took centre stage. Drawing on scientific evidence, including randomised controlled trials findings, epidemiological need and costeffectiveness modelling, the articles wove a story of need for PrEP as new HIV prevention, primarily for gay and bisexual men in the UK. In spite of ongoing global epidemiological need, UK newsprint framing of PrEP narrowed in on specific communities, excluding the possibility of wider user and went some way to close down the possibility of the intervention as something of use to the general population. Unlike new cancer treatments or other interventions that would impact the wider population, *PROUD* results were mobilised here to confirm the effectiveness of the intervention for gay men (Holt 2014). Our analysis focused exclusively on newsprint media, without reference to social media coverage of PrEP; however, the shift between community and clinician *to* clinician/scientist within this reporting reflects a closing down of who is creating the story of PrEP and how this might reflect and/or influence – or certainly frame – the imagined PrEP user in wider cultural narratives that affect policy (Treichler 1999).

Our analysis adds to a relatively small body of PrEP media analyses during a similar period (Card et al. 2019; Mowlabocus 2019; Jaspal and Nerlich 2017) by showing how scientific evidence was deployed to shape both the reliability of the intervention *and* to construct PrEP users. Jaspal and Nerlich (2017) investigate PrEP newsprint coverage during an overlapping period (2008–2015), illustrating the use of either highly positive or highly negative representations of PrEP through narratives of hope or risk. Looking explicitly at how PrEP is anchored and how this might affect uptake of PrEP, they argue that this polarisation could close down nuanced and realistic understandings of PrEP for potential users. Our analysis, incorporating coverage of PrEP one year after the *PROUD* findings, found that narratives of hope and risk were only part of the story; we found that intervention effectiveness (and certainty), epidemiological need and cultural narratives of responsible sexual practice played an important role in how PrEP users were configured. Mowlabocus’ (2019) explicitly considers how homonormativity is deployed during a period which incorporates coverage of the post-NHS England policy decision and highlights the persistence of well-rehearsed stereotypes of gay men. Findings show how these stereotypes were largely absent during the *post-PROUD* period, suggesting that the media deployment of PrEP evidence tempered – for a time – these familiar, homophobic tropes. While many of these articles – either explicitly or implicitly – explore PrEP in relation to LGBT identities and sexual practices, we argue that attention needs to be paid to the deployment of scientific evidence and its specific role in shaping who PrEP was for.

By mapping articles against key scientific events, we illustrate how reporting of particular scientific findings plays a key role shaping narratives around new public health interventions. We argue that this is an example of the processes of biomediatisation at work (Briggs and Hallin 2016). More than simply influencing users, the elements we have identified – the deployment of scientific narratives around end users, intervention effectiveness and epidemiological need – are constitutive of PrEP as an intervention for a ‘minority’ of the population, thereby shaping the boundaries of debate and the terms on which value, need and ultimately policy debates might be based. Noticeably absent within UK newsprint reporting were key global PrEP scientific events; the publication of World Health Organisation prevention guidelines, which recommended PrEP for men who have sex with men (WHO 2014) and the failed clinical results from other major PrEP trials with women (Rossi 2013) would also play a role in configuring potential PrEP users internationally. We suggest that this absence in UK newsprint is indicative of key events influencing UK policy and reflects the UK domestic PrEP agenda, rather than engaging or ignoring the wider global scientific context.

Ultimately, the PrEP user, configured as a cis-gendered gay and bisexual man, was consolidated during this time period. While gender (and sexuality) in pre-*PROUD* UK newsprint was non-specific and referenced a general population in the global epidemic, PrEP emerged as a specific intervention for gay and bisexual men. Gendered configurations of PrEP users have implications for how communities, health practitioners and policy makers engage with PrEP in real world settings. Indeed, this articulation of PrEP users could certainly be implicated in NHS England’s PrEP decision, which appeared to draw on underlying (or overt) notions of who is a deserving (and responsible) patient (Keogh 2008). This configuration of PrEP users, grounded in particular gender identities and sexual practices, would become apparent in subsequent reporting (Twocock 2016; Mowlabocus 2019). While the genuine PrEP need for gay and bisexual men should be highlighted, the possibility of other users was lost, or significantly diminished; this reinforces existing inequalities and reflects patterns observed through the HIV epidemic. However, we can currently see attempts to address and expand this particular gendered framing of PrEP users by activists in the UK (PrEPster 2018; Sophia Forum 2018).

### Strengths and limitations

This research has focused exclusively on newsprint media (including in print and online editions) and excluded social media coverage of PrEP which may have identified a more community-informed focus. We also limited our analysis to March 2016, thereby excluding responses to NHS England’s policy decision and subsequent high court battles. Whereas post-March 2016 reporting focused significantly on the rights and wrongs of the NHS decision, we were keen to explore how evidence was deployed and users configured in the lead up to this controversial decision. Our analysis is also specific to UK newsprint and does not chart differential patterning of wider global PrEP implementation. Nevertheless, a strength of our analysis is the focus on the ways that scientific reporting can open up or close down spaces in which potential PrEP users are imagined, and has implications for whether, and how, PrEP users may engage with and take up the interventions elsewhere.

## Conclusion

Scientific evidence, as reported in mainstream newsprint media, can play an important role in shaping the public health – or technological – imaginaries (Davis 2014) in relation to trust of an intervention and configuring potential end users. While scientific evidence can increase confidence in PrEP as well as other new public health technologies, these configurations can close down possibilities for other users, reinforce ideas of who is a deserving patient and play into wider concerns about the use of public resources for improving health and wellbeing. Following Triechler, we argue that there is a need to attend to language as a site in which the meanings of PrEP are constructed and determined. The PrEP story told in UK newsprint in the lead up to NHS England’s decision is only one of the many meanings of PrEP, and the role that it played in this decision is both unclear and beyond the scope of this paper. Nevertheless, charting the way in which scientific evidence and community voices in relation to public health need emerged during this period call attention to how media can craft and shape key issues in relation to public health, deserving publics and policy decisions. Our findings have implications for clinical and community practice in HIV prevention. Practitioners who work with women, trans communities and others who may benefit from PrEP should not only be aware of these media framings but will need to play an active role in responding to – and rebalancing – them to help identify and support those who may benefit from PrEP.

## Figures and Tables

**Table 1:** Broad thematic coding categories.

1.	What is the main focus of the article?
2.	What is the main tone of the headline?
3.	What is the main tone of the article?
4.	Who is making comments about PrEP?
5.	How is PrEP being framed?
6.	Interpretative comments on the form and structure of the article
7.	How does the article draw on scientific evidence?
8.	What are the reported criticisms of PrEP?
9.	What are the reported benefits of PrEP?

**Table 2:** Final sample of newsprint articles January 2012–March 2016.

Date	Newspaper	Headline
*Priming for PROUD*		
9 May 12	*Daily Mail*	First drug to prevent as well as treat HIV a step closer to approval
17 July 12	*Guardian*	US FDA approves first drug shown to reduce risk of HIV infection
17 July 12	*Guardian*	US approves pill that guards against HIV: Truvada to be available to people at extreme risk AIDS support group labels move ‘completely reckless’
17 July 12	*Daily Mail*	The first drug which can PREVENT HIV: pills reduce risk by up to 75% in at risk heterosexual couples
13 June 13	*Daily Mail*	New AIDS prevention pill could cut infection rates in IV drug users by 50%
10 February 14	*The Times*	Trial of pill to prevent HIV could lead to a risk in promiscuity
25 April 14	*Daily Mail*	More mixed-status couples conceiving children without protection as new treatments minimise the risk of infection
17 May 14	*Daily Mail*	Pressure grows on NHS to make new HIV wonder drug available for those at risk
17 May 14	*The Independent*	NHS urged to prescribe daily HIV pill to gay men
8 September 14	*Daily Mirror*	Revolution that could halt epidemic of HIV
30 September 14	*Guardian*	HIV protection in a pill for those at risk
17 October 14	*The Times*	Coming soon: the daily pill that can protect against HIV
17 October 14	*Daily Mail*	‘Wonder drug’ which can reduce HIV risk by 92% could be offered on the NHS
18 October 14	*Guardian*	Analysis: an HIV prevention pill is at hand, but will it be left untaken?
30 October 14	*The Independent*	Revolutionary new anti-HIV pill shown to work when taken ‘on demand’
*Post-PROUD*		
24 February 15	*The Independent*	HIV pill: the logic of paying £500 a month so gay men don’t have to wear condoms
24 February 15	*Telegraph*	Scientists hail daily pill that cuts HIV risk by 87%
25 February 15	*The Sun*	DOCS’ PLEA FOR HIV PILL ON NHS
25 February 15	*The Independent*	Scientists hail daily pill that protects against HIV infection
25 February 15	*The Independent*	The logic of paying £500 a month so gay men don’t have to wear condoms
25 February 15	*The Independent*	HIV pill: Scientists hail discovery of ‘game-changer’ that cuts the risk of infection among gay men by 86%
25 February 15	*Guardian*	Daily pill Truvada cuts spread of HIV by 86% study shows
25 February 15	*Daily Telegraph*	Before-and-after pill cuts HIV risk
25 February 15	*The Times*	Daily pill reduces HIV risk by 90%
25 February 15	*The Scotsman*	HIV game-changer as drug protects healthy gay men
26 February 15	*Daily Mail*	HEALTHY GAY MEN SHOULD BE GIVEN £440-A-YEAR HIV PILLS ON THE NHS’
26 June 15	*The Independent*	Pride in London: NHS to come under pressure to provide ‘miracle’ HIV prevention pill
27 June 15	*The Independent*	Pride hears calls for NHS to introduce HIV pill
10 October 15	*The Independent*	Daily pill that lowers HIV risk ‘could save NHS millions’
10 October 15	*The Independent*	NHS ‘cannot afford to ignore game-changing HIV drug’, experts say
11 October 15	*The Independent*	Cheap at the price; Editorial The NHS should fund prescription of a new AIDS treatment
11 October 15	*The Independent*	AIDS vanquished: a costly new pill promises to prevent HIV infection
24 October 15	*Daily Mail*	Could drugs to prevent HIV actually INCREASE the risk of infection by encouraging people not to use condoms?
16 November 16	*Daily Mail*	Once-a-day pill ‘DOES prevent HIV in the real world’: PrEP drugs ‘are effective in protecting health gay men from infection’
2 December 15	*The Herald*	HIV charity in call for NHS to prescribe drug
11 December 15	*Guardian*	The choice to take the HIV prevention pill has nothing to do with sluttiness
14 January 16	*The Times*	Fears HIV pill could encourage risk behaviour
14 January 16	*Telegraph*	Daily HIV pill for men ‘would prevent 10 000 new cases in UK by 2020’
14 January 16	*Daily Mail*	Daily pill to prevent HIV may be offered to thousands of men on the NHS after trials showed it could cut risks by 60%
25 February 16	*Daily Mail*	Gay man taking daily HIV prevention pill contracts resistant strain of the virus – in first recorded case of PrEP failing
21 March 16	*Daily Mail*	Could a monthly injection prevent HIV? Single shot of PrEP drugs is ‘as effective as taking pills twice a day’
25 March 16	*Guardian*	NHS England stalls plan for HIV prevention drugs
22 March 16	*The Times*	Pill to stop HIV will not get funding
22 March 16	*Daily Mail*	Charities slam NHS England and ‘U-turn’ over plans to roll out ‘HIV wonder drug’

**Table 3:** Overview of findings according to theme and time period.

	Ir/responsible citizens	Public health imperative
Priming for *PROUD* July 2012–January 2015	PrEP users as potentially irresponsible actors Risk of STIs/reduced condom use Mixed or unsupportive community responses PrEP effectiveness in ‘real world’ is uncertain	New infections globally with increased focus on vulnerable women and/or general population PrEP as expensive/costly
*Post-PROUD* February 2015–March 2016	PrEP users as responsible An absence of ‘risky’ sexual practices Limited or stable STIs PrEP will work/certainty of intervention	PrEP is a ‘game-changing’ intervention Gay & bisexual men in UK at most risk of HIV and in real need of new prevention tools Limited or no discussion of PrEP for other groups PrEP is a cost-effective intervention

## References

[R1] Altheide DL (2012). Qualiative Media Analysis.

[R2] Baral S, Poteat T, Stromdahl S, Wirtz A, Guadamuz T, Beyrer C (2013). Worldwide Burden of HIV in Transgender Women: A Systematic Review and Meta-Analysis. The Lancet Infectious Diseases.

[R3] Briggs C, Hallin D (2016). Making Health Public How News Coverage is Remaking Media, Medicine, and Contemporary Life.

[R4] Card K, Hawkins B, Mortazavi L, Gregory A, Ng KH, Lachowsky N (2019). Stigma, the Media, and Pre-Exposure Prophylaxis for HIV Prevention: Observations for Enhancing Knowledge Translation and Resisting Stigma in the Canadian Context. AIDS & Behavior.

[R5] Clarke AE, Mamo L, Fosket JR, Shim JK (2010). Biomedicalization: Technoscience, Health and Illness in the US.

[R6] Court and Tribunals Judiciary (2016). National Aids Trust -v- NHS England.

[R7] Davis M (2014). After the Clinic? Researching Sexual Health Technology in Context. Culture, Health & Sexuality.

[R8] Duffy N (2016). Journalism Union Condemns ‘Out-Dated Homophobic Tropes’ in PrEP Coverage. Pink News.

[R9] Epstein S (1996). Impure Science: AIDS, Activism and the Politics of Knowledge.

[R10] Hilton S, Hunt K, Langan M, Bedford H, Petticrew M (2010). Newsprint Media Representations of the Introduction of the HPV Vaccination Programme for Cervical Cancer Prevention in the UK (2005–2008). Social Science & Medicine.

[R11] Holt M (2014). Configuring the Users of New HIV-Prevention Technologies: The Case of HIV Pre-Exposure Prophylaxis. Culture, Health & Sexuality.

[R12] Jaspal R, Nerlich B (2017). Polarised Press Reporting about HIV Prevention: Social Representations of Preexposure Prophylaxis in the UK Press. Health.

[R13] Johnson E (2017). Gendering Drugs: Feminist Studies of Pharmaceuticals.

[R14] Keogh P (2008). How to Be a Healthy Homosexual: HIV Health Promotion and the Social Regulation of Gay Men in the United Kingdom. Journal of Homosexuality.

[R15] Kippax S, Stephenson N (2012). Beyond the Distinction between Biomedical and Social Dimensions of HIV Prevention through the Lens of a Social Public Health. American Journal of Public Health.

[R16] Lancet T (2016). UK PrEP Decision Re-Ignites HIV Activism. The Lancet.

[R17] McCallum K, Holland K (2017). To Drink or Not to Drink’: Media Framing of Evidence and Debate about Alcohol Consumption in Pregnancy. Critical Public Health.

[R18] Montgomery C (2012). Making Prevention Public: The Co-Production of Gender and Technology in HIV Prevention Research. Social Studies of Science.

[R19] Mowlabocus S (2019). What a Skewed Sense of Values’: Discussing PrEP in the British Press. Sexualities.

[R20] NAT (2016). Final PrEP HIV Drug Case Win for National AIDS Trust at Court of Appeal.

[R21] Neuendorf KA, Kumar A, Mazzoleni G (2006). The International Encyclopedia of Political Communication.

[R22] Nguyen V-K, Bajos N, Dubois-Arber F, O’ Malley J, Pirkle CM (2011). Remedicalizing an Epidemic: From HIV Treatment as Prevention to HIV Treatment is Prevention. AIDS.

[R23] Oudshorn N, Rommes E, Sinestra M (2004). Configuring the User as Everybody: Gender and Design Cultures in Information and Communication Technologies. Science, Technology, & Human Values.

[R24] Patterson C, Hilton S, Weishaar H (2016). Who Thinks What about e-Cigarette Regulation? A Content Analysis of UK Newspapers. Addiction.

[R25] Pickersgill M, Broer T, Cunningham-Burley S, Deary I (2017). Prudence, Pleasure, and Cognitive Ageing: Configurations of the Uses and Users of Brain Training Games within UK Media, 2005–2015. Social Science & Medicine.

[R26] PrEPster (2018). PREP4Women Facebook Live Event.

[R27] Press Gazette (2016). ABC figures: National press sees June Brexit vote boost in print and online.

[R28] Rosengarten M (2010). HIV Interventions: Biomedicine and the Traffic between Information and Flesh.

[R29] Rossi L (2013). Daily HIV Prevention Approaches Didn’t Work for African Women in the VOICE Study.

[R30] Seale C (2002). Media and Health.

[R31] Sophia Forum (2018). Women and PrEP.

[R32] Stansistreet M (2015). Specialist Journalists Shouldn’t Become an Endangered Species. The Guardian.

[R33] Treichler P (1999). How to Have Theory in an Epidemic: Cultural Chronicles of AIDS.

[R34] Twocock P (2016). The Daily Mail’s PrEP Story Was like Going Back to the Dark Old Days of the 80s. The Guardian.

[R35] Watney S (1987). Policing Desire: Pornography, AIDS and the Media.

[R36] WHO (2014). Consolidated Guidelines on HIV Prevention, Diagnoses, Treatment and Care for Key Populations.

[R37] Williams S, Seale C, Boden S, Lowe P, Steinberg D (2008). Medicalization and beyond: The Social Construction of Insomina and Snoring in the News. Health.

[R38] Young I, Davis M, Flowers P, McDaid L (2019). Navigating HIV Citizenship in the Treatment as Prevention Era. Health, Risk & Society.

[R39] Young I, Flowers P, McDaid L (2016). Can a Pill Prevent HIV? Negotiating the Biomedicalisation of HIV Prevention. Sociology of Health & Illness.

